# Deep Learning for Validating and Estimating Resolution of Cryo-Electron Microscopy Density Maps †

**DOI:** 10.3390/molecules24061181

**Published:** 2019-03-26

**Authors:** Todor Kirilov Avramov, Dan Vyenielo, Josue Gomez-Blanco, Swathi Adinarayanan, Javier Vargas, Dong Si

**Affiliations:** 1 Computing and Software Systems, University of Washington, Bothell, WA 98011, USA; tavramov@uw.edu (T.K.A.); dvnlo@uw.edu (D.V.); 2 Department of Anatomy and Cell Biology, McGill University, Montreal, QC H3A 0C7, Canada; josue.gomez-blanco@mcgill.ca (J.G.-B.); swathi.adinarayanan@mail.mcgill.ca (S.A.)

**Keywords:** computational structural biology, cryo-electron microscopy, deep learning, resolution validation

## Abstract

Cryo-electron microscopy (cryo-EM) is becoming the imaging method of choice for determining protein structures. Many atomic structures have been resolved based on an exponentially growing number of published three-dimensional (3D) high resolution cryo-EM density maps. However, the resolution value claimed for the reconstructed 3D density map has been the topic of scientific debate for many years. The Fourier Shell Correlation (FSC) is the currently accepted cryo-EM resolution measure, but it can be subjective, manipulated, and has its own limitations. In this study, we first propose supervised deep learning methods to extract representative 3D features at high, medium and low resolutions from simulated protein density maps and build classification models that objectively validate resolutions of experimental 3D cryo-EM maps. Specifically, we build classification models based on dense artificial neural network (DNN) and 3D convolutional neural network (3D CNN) architectures. The trained models can classify a given 3D cryo-EM density map into one of three resolution levels: high, medium, low. The preliminary DNN and 3D CNN models achieved 92.73% accuracy and 99.75% accuracy on simulated test maps, respectively. Applying the DNN and 3D CNN models to thirty experimental cryo-EM maps achieved an agreement of 60.0% and 56.7%, respectively, with the author published resolution value of the density maps. We further augment these previous techniques and present preliminary results of a 3D U-Net model for local resolution classification. The model was trained to perform voxel-wise classification of 3D cryo-EM density maps into one of ten resolution classes, instead of a single global resolution value. The U-Net model achieved 88.3% and 94.7% accuracy when evaluated on experimental maps with local resolutions determined by MonoRes and ResMap methods, respectively. Our results suggest deep learning can potentially improve the resolution evaluation process of experimental cryo-EM maps.

## 1. Introduction

There are three principle methods used for determining the 3D atomic structures of proteins; they include X-ray crystallography, Nuclear Magnetic Resonance (NMR) spectroscopy, and 3D cryo-electron microscopy (cryo-EM). The goal of these imaging techniques is to generate a high-quality and high-resolution, detailed protein macromolecule map that can be used in conjunction with other biochemical experiments and computational methods to generate a structural atomic model of the biomolecule. X-ray crystallography has been the gold-standard approach for solving protein structures for years [1]. Up to today, most protein structures with near-atomic or atomic resolutions have been determined by X-ray crystallography. However, large macromolecules that make up cell organelles such as ribosomes or those showing high degrees of flexibility and heterogeneity such as membrane proteins do not crystallize easily (or do not crystallize at all) and have proven to be a challenge for X-ray crystallography [2,3]. On the other hand, NMR can provide unique information on dynamics and interactions of proteins; however, atomic structure determination is restricted to small complexes with molecular weights of approximately <90 kDa. Both techniques typically require large amounts of relatively pure samples (on the order of several milligrams).

Cryo-electron microscopy is a form of transmission electron microscopy in which the studied sample is frozen in a thin layer of a noncrystalline form of solid water, termed amorphous ice, at cryogenic temperatures—generally that of liquid ethane. This technique is used extensively in structural biology to obtain the 3D structural information of macromolecular complexes at subnanometer or nanometer resolution depending on the technique used. The popularity of cryo-EM stems from the fact that it allows observation of biological specimens in their native environment, not stained or fixed in any way. This contrasts with X-ray crystallography, where the specimen needs to be crystallized, necessitating difficult and time-consuming procedures that typically place the biological samples into non-physiological environments, which can lead to functionally irrelevant conformational changes.

Single particle analysis (SPA) is a form of cryo-electron microscopy that allows acquisition of three-dimensional information for large and small macromolecules. Biological samples are processed in their native state, and thus captured in their native conformations. Furthermore, experimental samples need a lower concentration of protein than X-ray crystallography and NMR (micrograms instead of milligrams) [4,5]. In SPA, the idea of data collection is to make use of the fact that a macromolecule or particle occurs in multiple copies with (essentially) identical structure, and that the orientation of these particles is essentially random, ranging throughout the entire angular space with no major gaps. Then, instead of having to tilt the sample at various angles, in SPA, it is possible to take simultaneous snapshots of several molecular views. All these projections are then combined into a density map that depicts the molecule in three dimensions. This ideal situation rarely occurs and usually the macromolecule under analysis presents flexibility or heterogeneity. As a consequence, the resolution of cryo-EM maps is not usually homogeneous in magnitude and shows different values along the obtained map [6,7,8].

In the last few years, cryo-electron microscopy using single particle analysis (termed in the following as cryo-EM or 3DEM) method has gained much attention among structural biologists due to breakthrough advances that have now allowed this technique to resolve macromolecules at near-atomic resolutions similar to the ones provided by X-ray crystallography and NMR. Cryo-EM was first used to resolve large molecular complexes such as ribosomes [9,10] and viruses [11] with relatively good resolution. Significant advances in hardware and software [12,13] have made cryo-EM suitable for imaging smaller proteins and led to the publications of the first cryo-EM structures at resolutions around 3.0–3.5 Å (1 Å = 10${}^{-10}$ m) in the 2013-2014 timeframe [14,15,16,17]. Since then, the development of the cryo-EM method has undergone a resolution revolution, culminating in the 2017 Nobel Prize in Chemistry [18]. Today, published 3DEM maps have resolutions approaching atomic (1–2 Å) range for appropriate samples, and can be directly used to produce 3D protein models [19]. The current resolution record of cryo-EM rests at 1.62 Å resolution Electron Microscopy Data Bank (EMDB) [20] entry code: 9599).

### 1.1. The Unresolved Problem of Resolution and Map Validation in Cryo-EM

**Figure 1:**
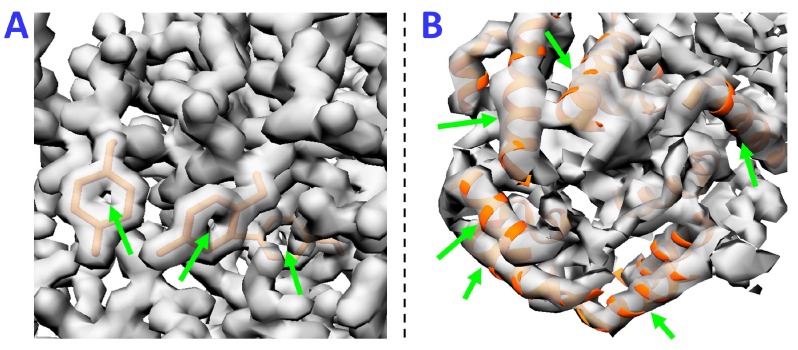
Approximate Resolution Assessment of cryo-electron microscopy (cryo-EM) maps. Simulated cryo-electron density maps of two example proteins at 2 Å and 6 Å, (**A**,**B**) respectively, illustrate how resolution can be estimated by the appearance of common structural elements. (**A**) density is thinned out in the center of aromatic rings, a feature typically observed in structures determined by X-ray crystallography at resolutions of 2 Å; (**B**) in density maps at medium (5–10 Å) resolutions, α-helices appear cylindrical in shape.

Optical resolution is traditionally evaluated by the smallest distance at which two objects can be distinguished from one another. However, this traditional resolution measure is not applicable for electron microscopy because obtained 3DEM maps are affected by noise that may distort this resolution metric. Nonetheless, resolution assessment and validation of cryo-EM maps is highly important for the correct interpretation and analysis of structural results. The concept of resolution in electron microscopy is not well defined and the currently used methods are subjective and not fully agreed on by the scientific community [21,22]. One approach to approximate resolution of a 3DEM density map is to compare it with an X-ray crystallographic reconstruction with a known resolution [23]. This method has a clear limitation that the X-ray crystallographic reconstruction has to exist to make such a comparison. The amount of detail in reconstructed EM maps can also be judged by the appearance of common structural elements, such as α-helices, β-sheets or aromatic rings (Figure 1). This approach is approximate and inherently subjective, and only applicable when the amount of detail in the reconstructed density map is high. When the amount of detail in computed density map is low, there are no external measures or resolved secondary structure elements by which resolutions can be judged.

Previous research has proposed computational and statistical methods for estimating the resolution of reconstructed 3DEM structures. Current practices include Differential Phase Residual, Q-Factor, Spectral Signal-to-Noise Ratio (SSNR) and Fourier Ring/Shell Correlation [22,24]. The most commonly used resolution measure today is the gold-standard Fourier Shell Correlation (FSC) procedure. The FSC calculates the similarity at different resolutions in frequency (Fourier) space between two independent 3D maps of the same protein, each calculated using one-half subset of the collected cryo-EM data [25]. The calculation produces an FSC curve as a function of the modulus of spatial frequency or resolution [22]. To derive a single resolution value from the FSC curve, a threshold criterion needs to be defined. The FSC threshold value is arbitrarily chosen as the point on the FSC curve at which the two reconstructions are considered consistent [25]. The threshold value and its interpretation remain a debated topic that has resisted a satisfactory solution. Some researchers have proposed various resolution thresholds (like FSC = 0.143, FSC = 0.5, and FSC = 1/3) as indicators of the resolution limit while others argue that fixed-valued FSC thresholds are not appropriate for all reconstructions due to the FSC dependency on the symmetry and size of the structure [26]. Additionally, because FSC uses two reconstructed maps computed from two halves of the data, the final resolution value varies depending on the processing method and whether the source data are split into two halves before or after alignment [22,27]. Therefore, many argue that FSC is a measure of the quality and consistency of the experiment and not a measure about the resolution and the physical details contained in the reconstructed map. Another issue of using the FSC as a tool to estimate the resolution is that this measure is invariant to any isotropic filtering of the entire dataset. Because the FSC method produces a single resolution value, it fails to account for locally variable resolution across the density map. Currently, there exist different approaches that can estimate the map resolution locally [6,7,8]. In the next section, we provide a short description of current approaches to estimate the map local resolution.

### 1.2. Map Local Resolution Determination

The first proposed approach to locally determine the map resolution was blocres [8]. In this approach, the resolution is locally estimated by means of the Fourier Shell Correlation, calculated from two independent maps within a moving window. Its main limitation is that the user has to define the size of the moving window and that the method requires two half maps. In 2014, Resmap was launched, which has represented the Gold-standard approach to obtain the map local resolution [6]. This approach determines the local resolution by detecting the best 3D sinusoidal wave that fits each map point above the noise level. This method requires a previous whitening process that is subjective and shows difficulties processing large volumes at least when used through Scipion platform [7]. More recently, a method called MonoRes was presented [7]. This approach is based on the Hilbert transform, which allows for obtaining the monogenic amplitude at each voxel for different resolutions. The resolution is locally obtained by comparing the monogenic amplitudes inside (macromolecule) and outside (noise) a provided 3D mask for each resolution within a defined resolution range. All these approaches require a considerable amount of processing time and in some cases their estimates are significantly different from one another.

### 1.3. Approach

The goal of our research is to overcome the limitations of the Fourier Shell Correlation method and the local resolution estimation methods by presenting deep learning approaches for resolution validation and estimation of cryo-EM density maps. Deep learning is an emerging new technology that has been applied in many disciplines of science and has been proven to be successful in image classification and image recognition tasks. Neural networks are a type of fully trainable deep learning models capable of capturing nonlinear relationships between inputs and outputs by automatically extracting high representative global and local information from image objects. Therefore, we would like to test the hypothesis that neural networks can be used to objectively quantify the resolution of 3D cryo-EM density maps. For this purpose, we use supervised learning techniques to construct neural network models capable of learning to distinguish electron density maps at high (<5 Å), medium (5–10 Å) and low(>10 Å) resolutions. We then use the trained models to evaluate simulated and experimental cryo-EM density maps. We further explore the feasibility of another type of neural network architecture, U-Net, for local resolution classification. Using a supervised learning technique and experimental maps only labeled by MonoRes and ResMap methods through Scipion method [28], we train a U-Net model to classify each map voxel into one of ten resolution categories. We then measure the agreement between the model predictions and the resolution estimates from MonoRes and ResMap.

## 2. Results

### 2.1. Model Metrics of DNN and 3D CNN Architectures

**Figure 2:**
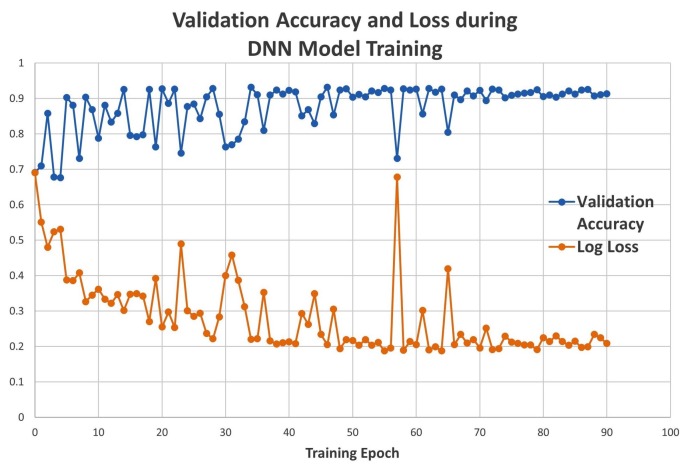
Dense artificial neural network (DNN) validation accuracy and loss.

The DNN model was trained, using simulated data, for 90 epochs (triggered by early stopping). The accuracy and the loss function value were recorded for the validation set at each epoch. The validation accuracy increased to around 92%, and the loss value decreased to 0.19 as the training progressed (Figure 2). After completion of the training stage, the performance of the DNN model was evaluated on a test data set containing simulated maps that were not included in the training process and were never seen by the trained model. The test accuracy of the DNN model was 92.73% on simulated maps.

**Figure 3:**
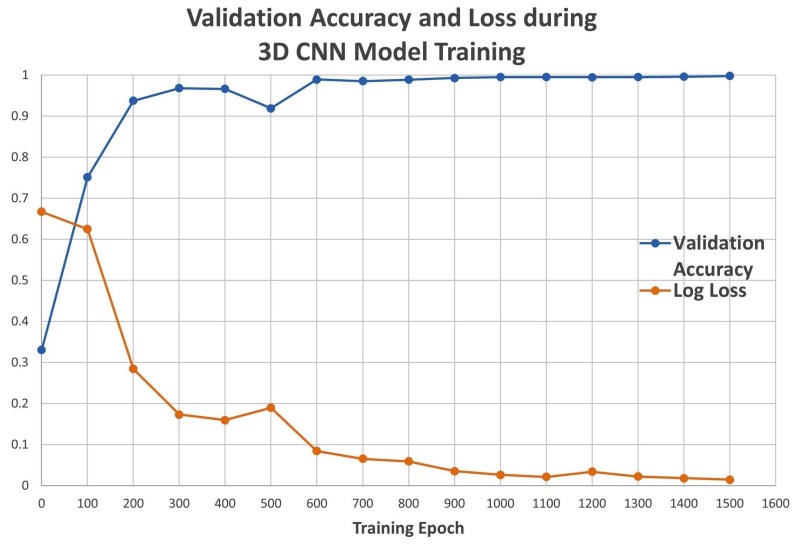
Convolutional neural network (CNN) validation accuracy and loss.

The training of the 3D CNN model was performed with simulated data and discontinued after 1500 epochs, at which point the optimization of the loss function was saturated. The validation accuracy and the loss function were recorded every 100 epochs during the training stage. The accuracy increased as training progressed and approached 100% at 1500 epochs, while the loss approached 0.01 (Figure 3). The test accuracy of the trained 3D CNN model was assessed on a data set containing simulated maps that were not included in the training process and were never seen by the model. The test accuracy of the 3D CNN model was 99.75%.

### 2.2. Performance of DNN and 3D CNN Models on Simulated Cryo-EM Density Maps at Different Resolutions

In addition to the 2.5 Å, 7.5 Å and 12.5 Å maps contained in the validation data set, the trained models were tested on an unseen dataset containing 28 different protein maps simulated at resolutions between 1.5 Å and 15.0 Å at each 0.5 Å increment. The protein protein data bank (PDB) ID was chosen at random for each resolution and care was taken to not include any structures contained in the training set. The 28 maps were downloaded from the Protein Data Bank and simulated with the “pdb2mrc” function [29] to a 64 × 64 × 64 box with 1 Å voxel spacing. The simulated maps were additionally preprocessed with the same normalization function as used for the training dataset. The 28 unlabeled maps were input to the pre-trained DNN and 3D CNN classifiers. The predicted resolution classes of all 28 maps are listed in Table 1.

Our models classified maps with similar resolutions as belonging to the same class. This experiment of classifying simulated maps with gradually variable resolutions identified the classification boundaries of the trained models. Classification boundary is the resolution at which the model changes its output from one class to another. The DNN model classified all maps with resolutions ≤4.0 Å as high, maps with resolutions between 4.5–8.5 Å as medium, and all other maps with resolutions ≥9.0 Å as low. The 3D CNN model classification boundaries between high-medium and medium-low resolution classes were at 4.5 Å and 8.5 Å, respectively. All maps predicted by the 3D CNN model followed these classification boundaries unlike the DNN model which had two outlying predictions; the 7.5 Å map was classified as low resolution and the 10.5 Å map was classified as medium resolution.

### 2.3. Performance of DNN and 3D CNN Models on Experimental Cryo-EM Density Maps

Thirty published cryo-EM maps were downloaded from the Electron Microscopy Data Bank (EMDB) [20]. The experimental maps were chosen based on their published resolution value; ten maps are claimed to have high resolutions ranging from 1.6 Å to 2.9 Å, ten had medium resolution between 6.0 Å and 8.0 Å, and ten had low resolution in the range from 11.0 Å to 14.8 Å. Table 1 lists the EM Data Bank (EMDB) IDs of the thirty experimental cryo-EM density maps and their claimed resolution values. The thirty experimental maps were preprocessed with the same normalization technique as used for the simulated maps. Because the trained models require an input of constant 64 × 64 × 64 size, an additional pre-processing step was applied to the experimental maps. The experimental maps had variable dimensions ranging from 45 to 432 voxels. For the maps with dimensions greater than 64, central cropping operation was applied to remove the outer parts of the maps and retain the central 64 × 64 × 64 region. In cases where a map had a dimension lower than 64, zero-padding was applied at the end to increase it to 64. The post-processed experimental maps were input to the DNN and 3D CNN models. Both models classified each experimental map as either high, medium or low resolution, and the results are included in Table 2.

We further measured the agreement between the unverified published resolution level and the resolution level predicted by the classification models. Table 3 and Table 4 show the confusion matrices for the DNN and 3D CNN models, respectively.

The confusion matrices count the number of maps that truly belong to each class and were predicted to belong to that class. The DNN model prediction and the published resolution match for all 10 high resolution maps, five medium resolution maps, and three low resolution maps, yielding a 60.0% combined agreement for all maps. The 3D CNN model prediction and the published resolution match for all 10 high resolution maps, four medium resolution maps, and three low resolution maps, yielding a 56.7% combined agreement for all maps. The DNN model classified 16 of the 30 maps as high resolution, 10 as medium resolution and only four as low resolution. The 3D CNN model classified 18 maps as high resolution, eight as medium resolution and four as low resolution.

To further assess the performance and predictive abilities of our classifiers, the confusion matrices were used to calculate the sensitivity, specificity, positive predictive value (PPV) and negative predictive value (NPV). The results included in Table 5 for the two multi-class classifiers are calculated according to the partial class membership method proposed by Beleitas et al. [30].

Sensitivity measures how well the classifiers recognize maps belonging to each resolution level while specificity measures the ability to recognize maps that do not belong to the given resolution group. PPV measures the probability that a given map truly belongs to the resolution group predicted by the model. NPV measures the probability that a map truly does not belong to a resolution group when the model predicts it does not belong to that resolution group. According to these results, the two classifiers have similar predictive capabilities except for the slightly better specificity and positive predictive value for high resolution maps of the DNN model over the 3D CNN model. Both are sensitive to high resolution maps but fail to recognize low resolution maps. The specificity for both classifiers is highest for low resolution maps and lowest for high resolution maps. For both classifiers, there is only a 50% certainty that a medium resolution prediction is correct as indicated by the PPV. However, the higher NPV for medium resolution suggests that our models can better classify maps that are not medium resolution.

### 2.4. U-Net Model Performance and Metrics on Experimental Cryo-EM Density Maps

Two separate U-Nets were trained, validated and evaluated on 13,444 and 1952 patches of input experimental cryo-EM density maps and their local resolution labels as determined by MonoRes and ResMap methods, respectively. In the data pre-processing stage, 14 and eight experimental cryo-EM density maps and corresponding MonoRes or ResMap label pairs were transformed and partitioned to smaller cubes (see Section 4). We designed the network to have an input layer of constant 16 × 16 × 16 size, which necessitated maps to be split into patches due to their variable dimensions. The 16 × 16 × 16 cubes, after the data processing steps, were split into distinct training, validation, and evaluation sets of input and label cubes. Post processed cubes were fed to the network during training or evaluation.

**Figure 4:**
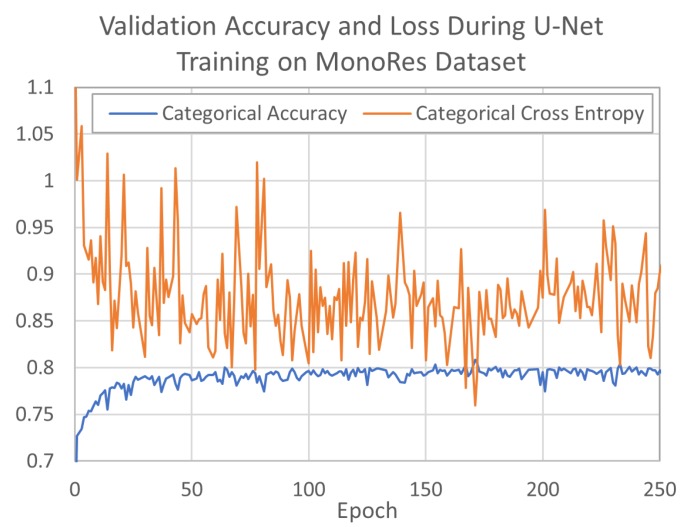
U-Net validation accuracy and loss when training on MonoRes data set.

**Figure 5:**
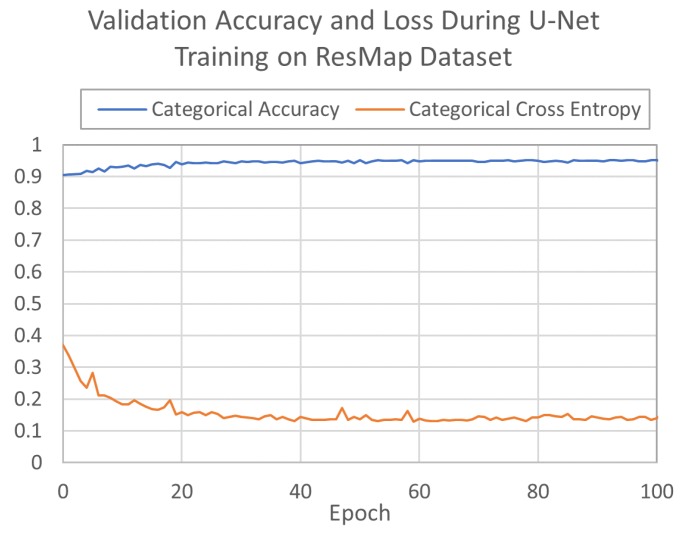
U-Net validation accuracy and loss when training on ResMap data set.

The two U-Nets were trained for 1172 and 1060 epochs on MonoRes and ResMap data sets, respectively. During training, the U-Net models learned to associate normalized cryo-EM density map voxel values with corresponding binned resolution class voxel values for the processed cubes. The networks optimized loss according to differences in predicted and label values with a categorical cross entropy loss function. Both networks converged quickly on training and validation sets; MonoRes converged in approximately 170 epochs (Figure 4), and ResMap converged in approximately 60 epochs (Figure 5). MonoRes settled on a validation loss of 0.87% and 79.9% accuracy while ResMap settled on validation loss of 0.19% and 95.0% accuracy. Training concluded after the network failed to further minimize validation loss for 1000 epochs.

The weights of the networks which produced the smallest loss values were saved and then used to evaluate the predictive capabilities of the models. Separate evaluation sets of experimental cryo-EM maps only, never seen by the networks, were used to evaluate the final accuracy of both networks. The MonoRes and ResMap datasets obtained evaluation accuracies of 88.3% and 94.7%, respectively. These reported accuracy values represent the mean likelihood of the network to predict a voxel resolution class value from an input cryo-EM density map voxel value. Accuracy was computed with a categorical accuracy function. Our reported values are the mean accuracy rate computed across all voxel predictions for the respective evaluation sets of the MonoRes and ResMap batches. Furthermore, a cross-comparison of the networks was conducted, by feeding the MonoRes evaluation set into the trained ResMap model, and likewise the ResMap evaluation set into the trained MonoRes model. Evaluation accuracies of the four combinations are presented in Table 6.

Results of the cross-comparison evaluations expose a fragility in the ResMap model to correctly predict voxel accuracy for the MonoRes evaluation set. Table 6 shows highest accuracies for ResMap model when evaluated with experimental maps labeled by ResMap (ResMap set). On the other hand, the lowest results are obtained when ResMap model is evaluated with maps labeled by MonoRes. These results require further investigation but suggest that the MonoRes method may provide more inconsistent estimations of local resolution than the ResMap approach. To fully understand the result and confirm this hypothesis, we will repeat this cross-comparison evaluation using balanced training and evaluation data sets.

## 3. Discussion

Both DNN and 3D CNN classification models were successfully trained, and minimized loss functions close to zero. The DNN model converged in 90 epochs, faster than the 1500 epochs needed to train the 3D CNN model. The 3D CNN model had higher prediction accuracy (99.75%) on the simulated test data set compared to the 92.73% of the DNN model. However, both the DNN and the 3D CNN models did not perform well on the experimental electron density maps. The two models had similar predictive capabilities as well as accuracies (60.0% for the DNN vs. 56.7% for the 3D CNN) when evaluating the experimental dataset of thirty published experimental maps. The large percentage of disagreement suggests that there are significant structural and volumetric differences between experimental and ideal (simulated) maps at each resolution level. In addition, cryo-EM maps usually show inhomogeneous resolution values; therefore, systematic use of a centered 64 × 64 × 64 cubic path for resolution assessment is not an accurate strategy. These observations also suggest that our approach to train on simulated maps was not adequate enough for the classification of experimental cryo-EM maps. Potential reasons of this issue are: (1) Simulated 3D electron density maps at different resolution levels from PDB models obtained by low-pass filtering do not accurately represent experimental cryo-electron microscopy maps. Note that these simulated maps are not affected by noise as they are obtained directly from atomic models; (2) Simulated maps present homogeneous resolutions as they were low-pass filtered to the same resolution value. Cryo-EM maps usually show inhomogeneous resolution values; therefore, different parts of the map exhibit different resolutions. To improve the accuracy of our deep learning approach, we decided to explore the feasibility of a supervised learning technique for local resolution classification with a U-Net architecture on MonoRes and ResMap experimental data sets. The preliminary results on this new approach show clear improvements in the accuracy of the model when evaluated with experimental cryo-EM maps, 88.3% and 94.7%. These results suggest training models locally with experimental cryo-EM maps is of great importance for obtaining high accuracy resolution estimations.

The DNN and 3D CNN models were trained to make coarse-grained classifications as only three resolution classes were considered, but the models could classify simulated maps with varying resolutions. The trained models consistently classified maps with similar resolutions to the same class. Our results also confirmed that comparable structural features exist in protein density maps at close resolutions. The structural features at resolutions less than 4–4.5 Å were picked up by the model as distinctly different than the features present at 5.0–8.5 Å density maps. The trained classification models also “learned” to recognize the features, or the lack thereof, in protein density maps at low (≥9.0 Å) resolutions. However, more than half of the thirty experimental maps were classified as high resolution: 16 by the DNN model and 18 by the 3D CNN model. The large number of reported medium- and low-resolution maps that were incorrectly classified as high resolution suggests that our models might be oversensitive to high-resolution features. However, both models classified as low resolution the same map (EMDB-5101) which was claimed to have 8 Å (medium) resolution in the EM Data Bank entry (see Table 1). This finding suggests a possibility that the author claimed resolution value may also be inaccurate.

The input layers of all proposed models require regular 3D voxel data with constant size which necessitated additional preprocessing (cropping and zero-padding) to transform the experimental 3D electron density maps into expected input dimensions. Proper voxelization of proteins into 3D density voxel maps was also required for the simulated maps used for training the models. Proteins can greatly vary in size and shape which makes determination of 3D voxel grid dimensions challenging. Selecting grid dimensions that are too small struggle to handle very large and/or irregular proteins and may result in maps that cut off a portion of the protein structure. Conversely, selecting very large grid dimensions may result in output that is unnecessarily voluminous with lots of zero intensity voxels, especially for density maps of small proteins. Additionally, the large grid size consumes a proportionally large amount of memory, which can slow down the performance and training of the neural network model. Our approach to address this issue was to use a 64 × 64 × 64 voxel grid and select relatively small proteins for the simulated dataset used for model training. Several simulated maps were visually inspected to confirm the 64 × 64 × 64 voxel box was large enough to fit the entire protein structure for the first two models. However, such manual inspection was impractical for all 12,671 proteins in the dataset leaving the possibility that some simulated maps were erroneously cut off.

Our third model augmented our approach but encountered similar or introduced new limitations. In the case of the data used for training the 3D Unet, we explored a different but still subjective size where large maps were split to smaller map segments of fixed dimensions. Moreover, this approach did not address the limiting nature of our imposed coordinate system. In addition, we encountered limitations in the capabilities of ResMap to compute the resolutions of larger map volumes which introduced variance between the MonoRes and ResMap data sets and limited the protein population we could ultimately train on [6]. Furthermore, our binning procedure may have introduced some degree of influence on feature values near bin margins, which, due to the preliminary nature of these results, has yet to be fully characterized. Nevertheless, we present these results as evidence to support the feasibility of neural networks to characterize local resolution from 3D cryo-EM density maps. In the future, we hope to address limitations of the U-Net model, as well as our other approaches.

Finally, the performance of supervised learning models is affected by the quality of training data. The simulated 3D maps were generated using a sampling rate of 1 Å/voxel while the experimental maps had varying voxel spacing. In addition, the training dataset was selectively chosen to include small single-chained proteins, while the experimental maps were of various large, multi-chain proteins. These limitations in the training set are probable explanations why our models did not perform that well on the experimental density maps as they performed on the simulated maps. Furthermore, the simulated maps filled the 3D volume of the 64 × 64 × 64 box and contained no noise outside of the molecule (density values were zero in the voxels where no atoms were present). However, experimental maps had varying box sizes not entirely filled by the molecule and the voxels outside of the protein atoms contained noise. The presence of noise in the experimental maps likely had a negative impact on the performance of the classifiers. It was also observed that the resolution values of the 30 experimental maps were determined by different resolution methods. Most of the published cryo-EM maps reported using the FSC method for calculating the map resolution, although with different FSC cut-off values (0.143 and 0.5). Other maps reported using a “Diffraction Pattern/Layerlines” method for determining the experimental resolution value [31]. There was no correlation between the predicted-published resolution agreement and the reported resolution method.

## 4. Materials and Methods

The 3D structures with exact atom coordinates of thousands of proteins have already been resolved and published in database archives [32]. In addition, there are image processing software tools [29,33] that can simulate fixed-size 3D electron density maps at a given resolution level from solved 3D atomic structures. To evaluate the hypothesis that deep learning models can extract and “learn” resolution features from 3D cryo-EM maps, datasets with simulated maps at three different resolution levels were created, pre-processed, and then used to train the neural networks. Two neural network architectures were explored: dense artificial neural network with nonlinearity (DNN), and 3D convolutional neural network (3D CNN). These deep learning models were first trained on thousands of simulated maps with their corresponding resolution label. The performance of the models was evaluated by comparing the predicted resolution category from the model with the known, simulated resolution of density maps that had not been used for training. In the second phase of our research, experimental 3D cryo-EM electron density maps were used for testing, and the models were evaluated based on the agreement between the published resolution value and the predicted value from the model. As an addendum, we explored the potential of supervised learning for localized resolution classification of experimental cryo-EM density maps. Using a 3D U-Net architecture, we adapted a model to classify voxels into one of ten half open, half closed resolution ranges. Input and label sets were collected, prepared, and two networks trained to discern corresponding label resolutions produced with MonoRes or ResMap software. We measure the likelihood of our networks to correctly predict an experimental voxel’s corresponding resolution label, and report the accuracies of our evaluated networks.

### 4.1. Data Collection and Data Pre-Processing

Solved 3D protein structures were downloaded from the publicly available Protein Data Bank (PDB) [32]. A query was used to select structures containing only single-chain proteins with molecular weights between 10 and 200 kDa (KiloDalton). The query returned around 47,000 results which were further reduced to 12,671 structures by removing models with more than 40% structural similarity. Using the EMAN2 software package and the function “pdb2mrc” [29], the selected 12,671 PDB structures were simulated to 3D electron density maps with 2.5 Å, 7.5 Å and 12.5 Å resolutions, corresponding to high, medium, and low resolution, respectively. The “pdb2mrc” function models each atom as a 3D Gaussian distribution, and the resolution value is the reciprocal of the half-width of that Gaussian distribution in Fourier space. The simulation box size was set to 64 × 64 × 64 and the sampling rate was 1 Å/voxel. The electron density values of all maps were linearly normalized between 0 and 1 to ensure uniform intensity ranges across all proteins. The 38,013 simulated normalized maps were uniformly and randomly split into training, validation and test sets with 60%-20%-20% ratio, respectively.

**Figure 6:**
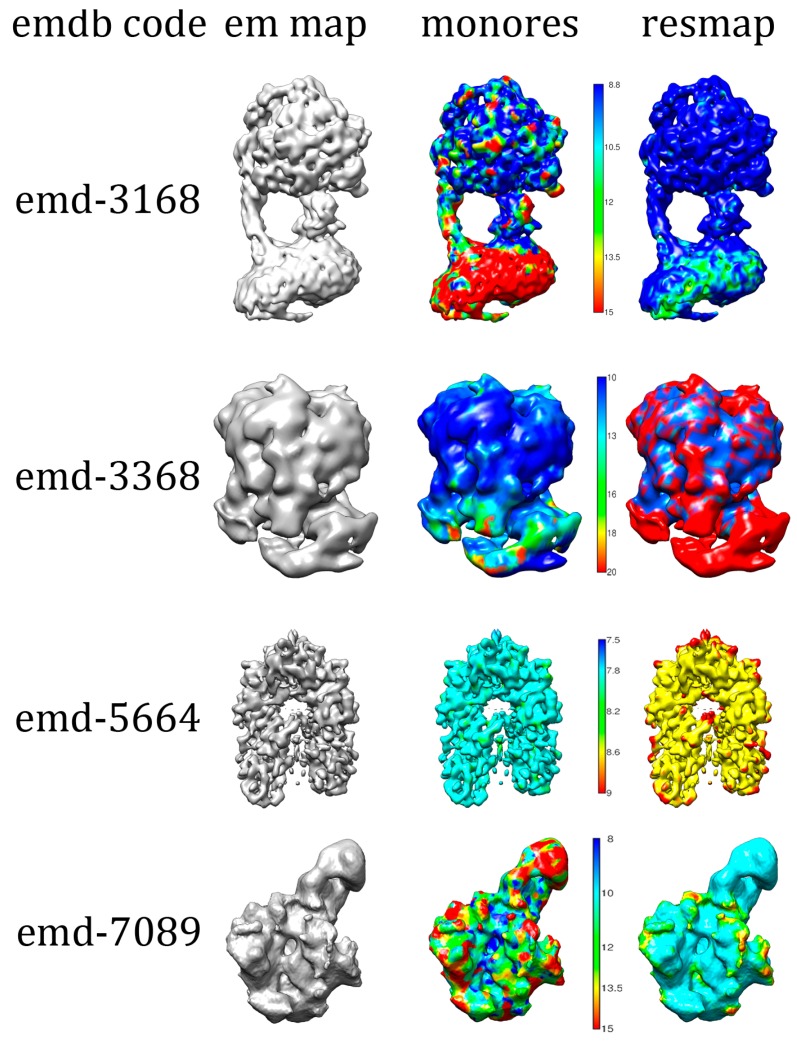
Examples of Electron Microscopy Data Bank (EMDB) structures and respective ResMap and MonoRes local resolution maps used to train the 3D U-Net model.

A second data set containing fourteen experimental cryo-EM density maps was obtained from EMDB as part of the localized resolution classification addendum. These maps underwent a transformation and partition protocol during a data preparatory stage to standardize features and morphology of experimental input maps and their corresponding MonoRes or ResMap label maps. In Figure 6, we show examples of some EMDB 3D structures and local resolution maps by ResMap and MonoRes approaches that were used to train the 3D U-Net model.

The MonoRes dataset contained labels for fourteen maps with the following EMDB IDs: 2984, 3098, 3168, 3296, 3368, 3619, 3691, 5653, 5664, 7089, 7341, 8194, and 9599. The ResMap dataset contained labels for eight proteins: 3098, 3168, 3340, 3368, 3691, 5653, 5664, and 7089. Map transformation involved normalization and binning of inputs and labels, respectively, as well as masking and padding. All inputs were feature normalized by min-max normalization into an inclusive range between zero and one. The labels were binned by classifying continuous voxel resolutions into one of ten classes: zero (0–2 Å), one (2–3 Å), two (3–4 Å), three (4–5 Å), four (5–6 Å), five (6–7 Å), six (7–8 Å), seven (8–9 Å), eight (9–10 Å), and nine (10–max() Å). Then, a binary mask, unique to each input and label, was applied to remove experimental noise from outside the protein’s structure. Finally, the maps were padded to the nearest power of two cube greater than or equal to the original shape of the input, label pair. We resolved odd sized dimensions by randomly appending a single element to the front or back of the dimension for an input, label pair. The maps were then partitioned by volume before entering the network. Transformed maps were divided into N cubes of shape (16, 16, 16) to form a rank 4 tensor of shape (N, 16, 16, 16). The empty cubes, those containing only zero values, were removed, and the remaining cubes aggregated into input and label repositories. The repositories were randomly partitioned into training (72%), validation (8%), and evaluation sets (20%) (Table 7). Partitioned sets were considered ready for processing in the network. Throughout the data preparatory stage, voxel-wise correspondence was validated and maintained by verification of a consistent shape and parallel treatment of inputs and labels. All of the data pre-processing steps were performed using Python 3.5.2. Visualization of protein models and electron density maps was done in a UCSF Chimera tool (version 1.12, University of California, San Francisco, CA, USA) [34].

### 4.2. Model Training

The neural network models were constructed and executed using the Python API of Tensorflow release version 1.5 software package (Google Brain Team, Google, Mountain View, CA, USA) on a Linux node with NVIDIA TITAN X (Pascal) (Nvidia Corp., Santa Clara, CA, USA) with 12 GB GPU memory. The training dataset of simulated maps was input into the models along with their known resolution class. Starting from default randomly initialized state, the models optimized a loss function that measures the probability error between the predicted and known labels during training. A non-exhaustive random search of different hyperparameters was performed to identify the set of loss functions, optimization algorithms, activation functions, learning rate, batch size and number of epochs that produced the overall smallest training error, highest validation accuracy, and fastest training time. The best performing DNN, 3DCNN, and 3D U-Net models are described in the next sections.

### 4.3. Dense Artificial Neural Network

**Figure 7:**
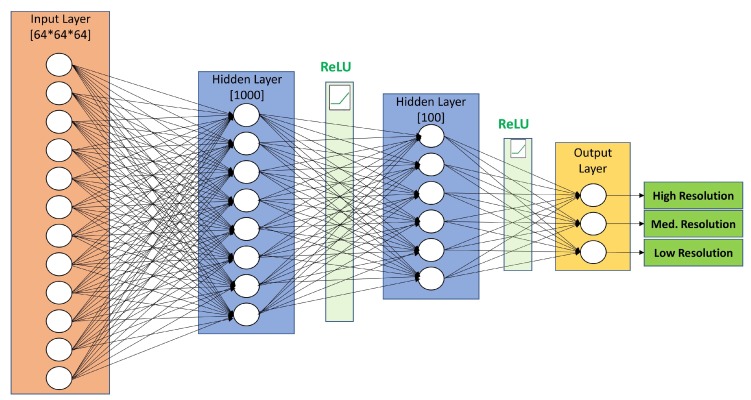
The fully connected neurons in the DNN Architecture and the application of non-linear mapping functions transform an input map into a single label corresponding to high, medium and low resolution.

Artificial neural networks (ANN) were first designed to model biological neural systems. They consist of multiple layers of neurons connected in an acyclic graph. In fully connected, dense artificial neural networks, all neurons in one layer are connected to all neurons in the next layer with an activation function applied along the connection. We construct one such network architecture for the resolution prediction of 3D EM maps. Our dense ANN contains an input layer and two hidden layers with 1000 and 100 neurons, respectively. The neurons in both hidden layers are followed by ReLU activation function. The purpose of the activation function is to build the nonlinear relationship between input and output. The final fully-connected output layer performed classification with three neurons, one for each resolution target class. The detailed configuration of the proposed DNN is provided in Figure 7.

The input layer with 64 × 64 × 64 neurons takes the voxels of a 3D map as input, multiplies the intensity values of each voxel with the weights of the 1000 neurons in the first fully connected layer, adds a bias, applies a ReLU mapping and feeds the output to the second hidden layer. The 100 neurons in the second hidden layer multiply, add a bias, and apply a ReLU nonlinearity to its input. Th three neurons in the output layer map probabilities to each class, with a softmax normalization. The DNN model was trained with Gradient Descent Optimizer and constant learning rate of 0.01. Sparse softmax cross entropy with logits was used as the training loss function. The training batch size was 150 and early stopping was implemented to stop training after 50 iterations with no progress in minimizing the loss function.

### 4.4. 3D Convolutional Neural Network

**Figure 8:**
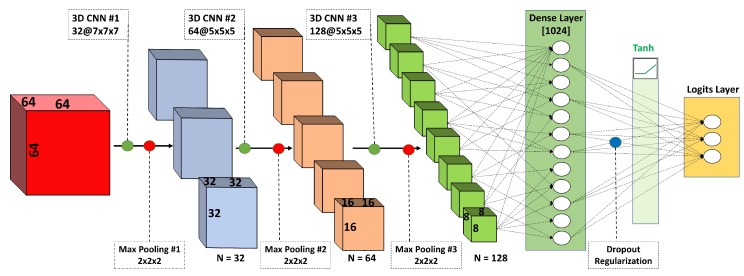
CNN architecture showing the layers of convolutional blocks of different size and the reduction of an input map to one of three output labels.

Stacked convolutional neural networks (CNNs) are currently the most popular model architecture for deep learning. This type of model architecture has been successfully applied in many image classification tasks. Convolutional networks have been shown to extract and learn higher-level features associated with a known label and then use these features to classify unseen images with unknown labels. Most of the previously published CNNs apply a series of filters to the raw pixel data of a 2D image. CNNs have achieved state-of-the-art performance on image and object recognition tasks [35,36]. In this paper, we first propose to use a 3D CNN architecture that uses three-dimensional convolutional filters applied to the three-dimensional electron density maps. Our 3D CNN contained stacked convolutional and pooling layers with different nonlinear activation functions, a fully connected dense layer and a logit output layer (Figure 8). The 3D CNN network maps an input 3D map to one of the three resolution classes (high, medium, low). The first convolutional layer (3D CNN#1) applies 32 7 × 7 × 7 filters. The second (3D CNN#2) and third (3D CNN#3) convolutional layers apply 64 and 128 5 × 5 × 5 filters, respectively. The first two convolutional layers are activated by an eLU activation function [37], and the third is activated by ReLU (not shown on figure). Zero-padding is used following each convolutional layer to preserve map dimensions. A max pooling operation with a 2 × 2 × 2 filter and stride of 2 is applied after each convolutional layer to downsample (reduce by half) the map data size. Dense layer with 1024 neurons activated by Tanh function and dropout regularization (rate = 0.4) performs classification on the features extracted by the convolutional and max pooling layers. The “logits layer” maps class probabilities with a softmax normalization and outputs one of the three resolution classes (0—high, 1—medium, 2—low). The 3D CNN model was trained with Gradient Descent Optimizer and learning rate of 0.01. Sparse softmax cross entropy with logits was used as the training loss function. The training batch size was 75.

### 4.5. 3D U-Net Architecture

**Figure 9:**
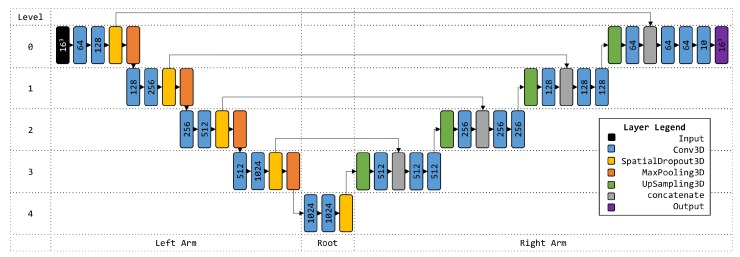
3D U-Net Architecture. Filter counts are shown on Conv3D layers. Element dimension is shown for Input and Output layers.

The U-Net is a convolution neural network architecture developed for biomedical image segmentation [38] and later expanded to volumetric segmentation [39]. Here, we apply the 3D U-Net architecture to classify local resolution of cryo-EM density maps in a supervised training approach. Our model consisted of an input layer, sequential layers grouped by level and arm, and an output (logits) layer. A detailed visualization of the network is provided in Figure 9.

The input layer accepted a batch of 32 16 × 16 × 16 categorical cubes with shape (32, 16, 16, 16, 1). Inputs propagated through layers on levels and arms of the network. Each level on the left arm consisted of Conv3D, Conv3D, SpatialDropout3D (0.5 dropout), and MaxPooling3D layers. Conv3D layers used various filter sizes (64, 128, 256, 512, and 1024), a kernel of size three cube, non-linear activation (ReLU), same padding, and He initialization. MaxPooling3D layers used a pool size two cube and a size two stride. The root group consisted of Conv3D, Conv3D, and SpatialDropout3D (0.5 dropout) layers. Conv3D layers in the root group were configured to the same parameters as those on the left arm. Layer groups from level three to zero on the right arm consisted of UpSampling3D (size two cube), Conv3D, concatenate, Conv3D, and Conv3D. The first Conv3D layer of each level group of the right arm used a kernel size two cube. Otherwise, the same parameters as those on the left arm were used for all Conv3D layers on the right arm. A concatenate layer joined SpatialDropout3D and the first Conv3D outputs from the left and right arms, respectively, of each level. The network terminates with an output Conv3D layer configured with ten filters for the ten classes, a kernel size 1 cube, softmax activation, the same padding, and He initialization. The U-net was optimized with an Adam optimizer and computed loss according to categorical cross entropy. An early stopping callback was implemented to stop training after 1000 iterations without further minimization of loss.

## 5. Conclusions

We have demonstrated that deep learning models can learn resolution patterns from cryo-EM density maps. Of the three models presented in this paper, two predicted the resolution of simulated maps, as either high, medium or low, with high accuracy and precision. The three-class classifiers were further shown to correctly group together simulated maps with similar resolutions. Using experimental cryo-EM density maps, the two models were shown to have specificity in the 60–95% range, although sensitivity was lacking, especially for medium- and low-resolution maps. The third model predicted local resolution of experimental 3D cryo-EM density maps. This U-Net model achieved greater predictive accuracy during evaluation on experimental maps than either of the preceding approaches. These results suggest that deep learning techniques can be used to validate resolutions of 3D cryo-EM density maps. More experiments are necessary to explain why the resolution prediction of experimental cryo-EM maps had low and variable accuracies. Future work will attempt to improve the resolution prediction of experimental maps and expose the variance in network accuracies for MonoRes and ResMap data sets. Central cropping and zero-padding may affect prediction results; therefore, other sizing approaches will need to be investigated. Further work is required to design models that are agnostic to different input sizes. Our research group is currently investigating the use of the PointNet architecture [40] for the resolution classification of 3D cryo-electron microscopy density maps. The PointNet neural network works with point clouds representing 3D geometry as input, thus avoiding the problem of selecting a rigid voxel grid size. The PointNet model has been shown to perform 3D object classification with better performance than models based on volumetric 3D input, and we hypothesize that it can achieve better results than the ones presented here. Future deep learning models may also benefit from using a more representative training data, sampled at different voxel spacings that more closely match the experimental maps in the EM Data Bank.

## Figures and Tables

**Table 1:** Classification results on simulated maps with varying resolution for dense artificial neural network (DNN) and convolutional neural network (3D CNN).

Simulated Resolution	DNN	3D CNN	Simulated Resolution	DNN	3D CNN
1.5 Å	High	High	8.5 Å	Medium	Medium
2.0 Å	High	High	9.0 Å	Low	Low
2.5 Å	High	High	9.5 Å	Low	Low
3.0 Å	High	High	10.0 Å	Low	Low
3.5 Å	High	High	10.5 Å	Medium	Low
4.0 Å	High	High	11.0 Å	Low	Low
4.5 Å	Medium	High	11.5 Å	Low	Low
5.0 Å	Medium	Medium	12.0 Å	Low	Low
5.5 Å	Medium	Medium	12.5 Å	Low	Low
6.0 Å	Medium	Medium	13.0 Å	Low	Low
6.5 Å	Medium	Medium	13.5 Å	Low	Low
7.0 Å	Medium	Medium	14.0 Å	Low	Low
7.5 Å	Low	Medium	14.5 Å	Low	Low
8.0 Å	Medium	Medium	15.0 Å	Low	Low

**Table 2:** Classification results of the 30 experimental cryo-electron microscopy (cryo-EM) density maps with dense artificial neural network (DNN) and convolutional neural network (3D CNN).

EMDB ID	Resolution	DNN	3D CNN	EMDB ID	Resolution	DNN	3D CNN
8221	1.6 Å	High	High	5751	8.0 Å	High	Medium
2984	2.2 Å	High	High	3340	7.2 Å	Medium	Medium
2945	2.9 Å	High	High	5101	8.0 Å	Low	Low
6342	2.5 Å	High	High	3168	7.4 Å	Medium	Medium
8218	2.3 Å	High	High	5653	7.3 Å	High	High
8222	2.5 Å	High	High	7089	13.2 Å	Low	Low
8077	1.75 Å	High	High	1601	14.1 Å	High	High
8217	1.8 Å	High	High	6013	14.8 Å	Medium	Medium
8219	2.5 Å	High	High	3368	13.0 Å	Medium	Medium
6313	2.5 Å	High	High	2862	12.5 Å	Low	Low
3311	6.7 Å	Medium	High	3098	11.0 Å	Medium	Medium
5664	7.8 Å	High	High	7471	12.5 Å	Medium	High
6410	7.8 Å	High	High	1711	13.0 Å	Low	Low
4047	6.0 Å	Medium	High	5169	11.0 Å	Medium	Medium
1674	6.0 Å	Medium	Medium	1981	14.2 Å	High	High

**Table 3:** DNN confusion matrix.

DNN Confusion Matrix		Predicted Resolution		
		High	Medium	Low
Published Resolution	High	10	0	0
	Medium	4	5	1
	Low	2	5	3

**Table 4:** CNN confusion matrix.

3D CNN Confusion Matrix		Predicted Resolution		
		High	Medium	Low
Published Resolution	High	10	0	0
	Medium	5	4	1
	Low	3	4	3

**Table 5:** Performance measures of the classifiers including positive predictive value (PPV) and negative predictive value (NPV).

		Sensitivity	Specificity	PPV	NPV
DNN Model	High	100.00%	70.00%	62.50%	100.00%
	Medium	50.00%	75.00%	50.00%	75.00%
	Low	30.00%	95.00%	75.00%	73.10%
CNN Model	High	100.00%	60.00%	55.60%	100.00%
	Medium	40.00%	80.00%	50.00%	72.70%
	Low	30.00%	95.00%	75.00%	73.10%

**Table 6:** U-Net model and evaluation set accuracies.

U-Net Evaluation Accuracies		Model	
		MonoRes	ResMap
Set	MonoRes	88.3%	59.4%
	ResMap	88.9%	94.7%

**Table 7:** Input and label cube counts for processed batches.

Set	MonoRes	ResMap
Train	9670	1400
Validation	1080	159
Evaluation	2694	393
Total	13,444	1952

## Abbreviations

The following abbreviations are used in this manuscript:

NMR	Nuclear Magnetic Resonance
cryo-EM	Cryo-Electron Microscopy
SSNR	Spectral Signal-to-Noise Ratio
FSC	Fourier Shell Correlation
SPA	Single Particle Analysis
DNN	Dense Neural Network
CNN	Convolutional Neural Network
PDB	Protein Data Bank
EMDB	Electron Microscopy Data Bank
kDa	KiloDalton
ReLU	Rectified Linear Units
